# Training in Capsule Endoscopy: Are We Lagging behind?

**DOI:** 10.1155/2012/175248

**Published:** 2012-04-11

**Authors:** Reena Sidhu, Mark E. McAlindon, Carolyn Davison, Simon Panter, Olaf Humbla, Martin Keuchel

**Affiliations:** ^1^Gastroenterology Hepatology Unit, Royal Hallamshire Hospital, Glossop Road, Sheffield S10 2JF, UK; ^2^Department of Gastroenterology, South Tyneside District Hospital, Tyne and Wear NE34 OPL, UK; ^3^Department of Internal Medicine, Bethesda Krankenhaus Bergedorf, Hamburg, 21029, Germany

## Abstract

Capsule endoscopy (CE) is a new modality to investigate the small bowel. Since it was invented in 1999, CE has been adopted in the algorithm of small bowel investigations worldwide. Reporting a CE video requires identification of landmarks and interpretation of pathology to formulate a management plan. There is established training infrastructure in place for most endoscopic procedures in Europe; however despite its wide use, there is a lack of structured training for CE. This paper focuses on the current available evidence and makes recommendations to standardise training in CE.

## 1. Introduction

Capsule endoscopy has revolutionised the way gastroenterologist image the small bowel. Since its approval by the Food and Drug Administration (FDA) in 2001, the indications for its use have expanded widely. There have been European, American, British guidelines among others on the use of CE [1–5]. Despite this worldwide expansion on the use of CE, there remains a lack of accepted standardized credentials for physicians who provide a CE service. There is also a lack of structured training for trainees who wish to undertake CE compared to other forms of gastrointestinal endoscopy. With such rapid expansion in uptake of this new modality comes the inherent need to develop diagnostic knowledge, skill, and competence assessments for CE.

## 2. Reading a Capsule Endoscopy Video

CE provides approximately 8–11 hours of small bowel footage, depending on the CE diagnostic system being used [6]. There are several prototypes of CE currently available on the market including the PillCam SB (Given Imaging, Yoqneam, Israel), the Endocapsule (Olympus, Tokyo, Japan), and the MiroCam (Intromedic, Seoul, Republic of Korea). Small studies making comparisons between these devices have shown no real difference between them [7–9]. The current available software allows the reader to visualise the images in a single, double, or quad views at rates of five to forty images per second. Images can be saved as thumbnails with annotations. On most softwares, there is a “suspected blood indicator” that identifies red pixels [10] which helps direct the reader to certain frames with likely pathology. There is also localization software that provides some estimate on the location of the capsule within the small bowel. The average reading time varies between 30 and 120 minutes depending on the small bowel transit, quality of images, and the experience of the reader [11].

## 3. Different Requirements for Training in Capsule Endoscopy

Numerous studies have compared CE reading between nurses, gastroenterology trainees/endoscopy fellows, and medical students [11–19]. Significant interobserver variation in reporting occurs even among experienced capsule endoscopists [16, 17]. Whilst studies have shown that the agreement on identification of landmarks and pathology is greater among experts compared to gastroenterology trainees [15, 16], there is no defined number of capsule endoscopies that would signify competence. However, prior endoscopic experience has been shown to enable trainees to interpret CE videos more accurately compared to medical students [19].

A number of studies have also identified a role for nurses in CE reporting as physician extenders [11, 12, 14, 20]. The published studies have shown that although nurses are more likely to identify additional insignificant findings, no serious pathology is missed compared to physician experts. More recently, one blinded trial (abstract) which compared the reading of CE between an experienced nurse and a doctor also found no significant difference in diagnostic yield and management advice given in CE reporting [21].

Training on small bowel CE has also been shown to be helpful in the interpretation of colon capsule endoscopy (CCE) images; however, on its own, it is deemed insufficient. In one of the first trials, CCE videos were read by physicians with extensive experience in small bowel CE. However, technicians specifically trained on CCE had a higher diagnostic yield during a separate reading [22]. As a consequence, physicians participating in a large subsequent multicenter study on CCE had to successfully complete test videos before starting enrolment [23].

## 4. Training: Setting the Scene

A structured training programme exists for the majority of gastrointestinal (GI) endoscopic procedures worldwide. In the UK, the Joint Advisory Group (JAG) has a minimum number of endoscopic procedures and set criteria for trainees to undertake prior to being deemed competent in each modality [24]. The evaluation of competence is also assessed by a minimum of two trainers for verification after completion of GI endoscopic portfolio for upper and lower GI endoscopy. In most other countries in Europe, a similar approach is adopted to ensure trainee competence.

Despite the limited evidence available on training in CE, in 2005 the American Society of Gastrointestinal Endoscopy (ASGE) recommended that training performed outside a GI fellowship should include the completion of a hand-on course with a minimum of 8 hours of continuing medical education, followed by review of the first 10 complete cases by a credentialed capsule endoscopist [25]. American guidelines for endoscopic training in routine procedures within a fellowship define 25 capsule endoscopy studies as a threshold for assessing competence (Gastroenterology Core Curriculum, third edition, 2007, jointly published by American Association for the Study of Liver Diseases (AASLD), American College of Gastroenterology (ACG), AGA Institute and American Society for Gastrointestinal Endoscopy (ASGE)).

In Britain, training in CE is not a mandatory requirement of specialist training, and many trainees receive no training at all in this field. A survey of trainee gastroenterologists highlighted that while they do request CE procedures for their patients, only 13% had ever had the chance to report a study [19]. The survey also revealed that 88% of trainees around the country were interested in learning about CE and 40% would consider becoming future CE service providers. Although there is interest evident from trainees, access to capsule services and in-house training is currently not universal. Furthermore, 45% of GI units in the UK routinely offer CE and more than 90% of UK gastroenterologists currently refer for capsule endoscopy [26]. This intense penetration of CE into daily practice clearly warrants standards for training and assessing competency. This unsatisfactory situation is a reflection for most other European countries as well.

## 5. Methods of Training

There have been numerous studies which have looked at methods of training in GI endoscopy. Studies using computer-based or virtual simulator models in upper GI endoscopy [27] and colonoscopy [28, 29] have shown to be beneficial with improved performance at endoscopy. The Erlangen Endo-Trainer with biological specimens from pigs has been adopted as a method of training in some centres in Germany with improvement in learning curves [30]. Apart from standard endoscopy and ERCP, this method has also been adopted for training in double balloon enteroscopy [31].

Few studies have addressed how best to train in CE. Whilst the literature suggest that prior endoscopic experience is helpful in CE reporting [19], the training required for CE is vastly different to the technical competence and hands-on training required for flexible sigmoidoscopy or colonoscopy. Capsule endoscopy requires a skill set based on observation, recognition, and interpretation of significant findings from computer images with appropriate management advice.

**Figure 1:**
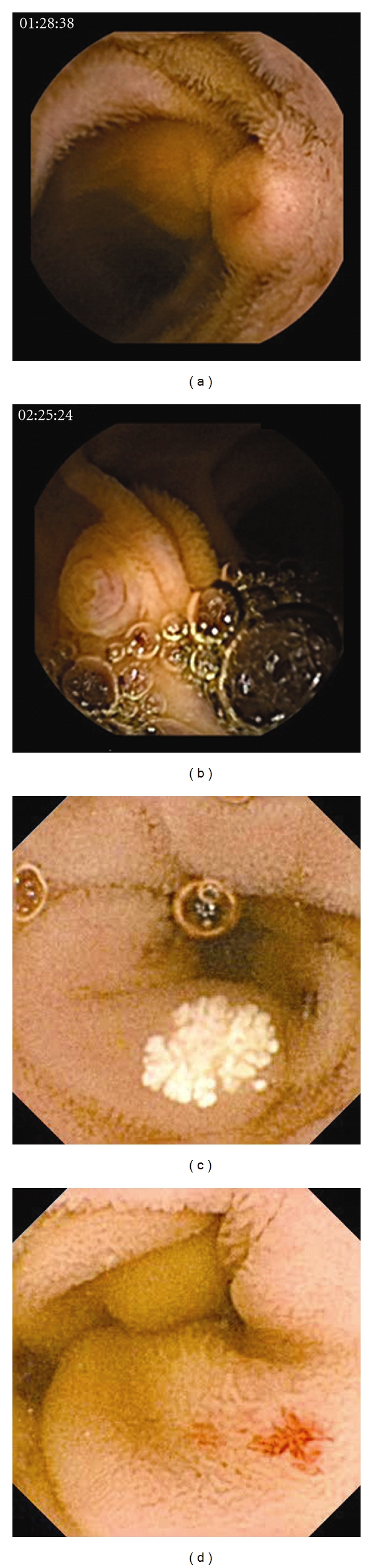
(a–d) Still images from test videos for evaluation of competency gained during formal beginners courses on capsule endoscopy. (a) Submucosal tumor (carcinoid), (b) normal papilla, (c) focal lymphangiectasia (variant of normal), and (d) angiectasia.

This requirement for training in CE has been incorporated into formal training courses in the United States and Europe, by providing hands-on training with a computer workstation for two or three delegates each. Although courses differ throughout Europe, the basic principles include hands-on training with a significant amount of time spent on real cases or case sequences. Topics covered in the courses generally include practical use of the software, anatomical landmarks, and diseases causing midgastrointestinal bleeding, as well as inflammatory and tumorous lesions of the small bowel. Many of the courses are partially sponsored by one of the manufacturers of CE. As principles for the clinical application of CE are independent from the capsule type, course curricula are almost identical irrespective of the software. However, for consistency of training, only one system is used during hands-on training on any single course in Europe. The American Society for Gastrointestinal Endoscopy (ASGE) offers split courses, providing training either with the Given Imaging or the Olympus System. Most of the European courses consist of two days: day one for beginners and day two for advanced training. Preliminary, unpublished data on several European courses have showed a significant improvement in the ability to classify type and relevance of small bowel findings, either pathology or variants of normal as shown in the CE video images (Figure 1) after attending a formal beginners course.

Books are a well-established source of education. There are books available on CE, focusing on a practical introduction to the method [32], on a comprehensive collection of images [33], or on the clinical context [34]. Accompanying DVDs with video clips [32, 34] may improve visual understanding.

Web-based or e-learning is a relatively new method which is fast becoming a valid educational method of postgraduate training within a range of medical specialities [35]. Postgate et al. assessed the utility of a computer-based CE lesion recognition learning module on 28 trainees with varying experience [36]. The trainees in the study demonstrated a significant improvement in lesion recognition skills after a dedicated computer-based training module, which consisted of video clips of normal anatomical appearances, incidental and pathological findings, and learning objectives and integrated feedback within multiple-choice questions [36]. The same group have also used an animal-based model in tandem with CE to assess the rate of polyp detection [37]. Although endoscopic experience was helpful, larger polyps, which are the most clinically relevant, tended to be the least accurately sized even by CE experts and experienced endoscopists [37]. Another model using pearls of different sizes in an animal gut visualized by CE was systematically undersized by students and by experts. However, experts with experience of more than 400 CE tended to be more precise, suggesting a continuing learning curve even after performing many examinations [38]. In a comparative multicenter trial on capsule videos segments, the poorest interobserver agreement was found for estimating the size of lesions [39].

Hence in the published literature there remains a paucity of evidence on how best to train and how much of training is required (learning curve) to achieve competence. However, studies on interobserver agreement have shown that correct detection and classification of polyps and ulcers seems more difficult than for angiectasis or active bleeding. This could provide the basis for selecting topics to be dealt with in more detail during courses.

Presently, formal training courses dedicated specifically to colon capsule endoscopy (CCE) are offered only in a few European countries such as in Spain. However, most advanced courses include an introduction to e-principles of CCE and some hands-on training on CCE cases.

## 6. Recommendation for Training in Capsule Endoscopy

Training in CE needs to be standardised and aligned with other forms of endoscopy training. At present, a small number of hands-on computer-based training courses are already established in the US, Europe, and the UK. The UK CE training programme, dual endorsed by both British and American GI Societies, currently provides training at beginner and advanced levels.

A core curriculum for CE is currently being established. The core curriculum should define competencies, learning outcomes, and assessments relating to CE. This should include assessment of the patient, the CE procedure, equipment, prereading, diagnosis, and reporting with management advice. Managing complications and medicolegal aspects should also be encompassed in the curriculum.

This can serve as a basis for national regulations and guidelines of endoscopic or gastroenterology societies. Training standards with competency measures should be set using formative and summative assessments, which could be carried out locally. Finally a formal framework for accreditation in CE for doctors and nurses should be established in a number of CE certified training centres or incorporated into established CE training programmes, in conjunction with national endoscopic bodies.

The degree of competency requested will depend on the expected role of the trainee after completing the curriculum. For instance, in some European countries there is a role for nurses in prereading, but in most countries, nurses will not be allowed by legal regulations to finally diagnose and report a video capsule study.

This formal structured process would in turn help formalise quality assurance of capsule endoscopy service development, practice, and training.

## 7. Learning Objectives and Practice Points

- Capsule endoscopy is a noninvasive modality to investigate the small bowel.
- Reporting a CE video requires identification of landmarks, interpretation of pathology, and formulation of appropriate management advice.
- There is established training infrastructure for most forms of endoscopy across Europe for trainees.
- Despite its wide use, there is a lack of structured training for CE.
- Prior endoscopic experience is beneficial in CE reading.
- Web-based CE learning has been proven to be useful in demonstrating an improvement in lesion recognition.
- A structured CE training programme is required with formal accreditation in CE.

## Abbreviations

CE: Capsule endoscopy
UK: United Kingdom
GI: Gastrointestinal
JAG: Joint advisory group
CCE: Colon capsule endoscopy
ASGE: American Society of Gastrointestinal Endoscopy.
